# Cell- Cell Transmission of VSV-G Pseudotyped Lentivector Particles

**DOI:** 10.1371/journal.pone.0074925

**Published:** 2013-09-10

**Authors:** Amy M. Skinner, Santhosh Chakkaramakkil Verghese, Peter Kurre

**Affiliations:** 1 Departments of Pediatrics, Papé Family Pediatric Research Institute, Oregon Health & Science University, Portland, Oregon, United States of America; 2 Department of Cell & Developmental Biology, Oregon Health & Science University, Portland, Oregon, United States of America; George Mason University, United States of America

## Abstract

Many replicating viruses, including HIV-1 and HTLV-1, are efficiently transmitted from the cell surface of actively infected cells upon contact with bystander cells. In a previous study, we reported the prolonged cell surface retention of VSV-G replication-deficient pseudotyped lentivector prior to endocytic entry. However, the competing kinetics of cell surface versus dissociation, neutralization or direct transfer to other cells have received comparatively little attention. Here we demonstrate that the relative efficiency of cell-cell surface transmission can outpace “cell-free” transduction at limiting vector input. This coincides with the prolonged half-life of cell bound vector but occurs, unlike HTLV-1, without evidence for particle aggregation. These studies suggest that cell-surface attachment stabilizes particles and alters neutralization kinetics. Our experiments provide novel insight into the underexplored cell-cell transmission of pseudotyped particles.

## Introduction

Cell-cell transmission plays an important role in the life cycle of replicating viruses where direct surface transfer can represent a more efficient mode of spread, compared with cell-free infection [1,2]. Surface attachment of particles has been shown to modulate infectivity and provide a reservoir for viral passage between cells [3–6]. For example, while cell-free vesicular stomatitis virus (VSV) is rapidly inactivated in circulation, cell surface retention offers temporary protection from neutralization [7–9]. Virus particles bound to the cell surface not only gain biological advantages of altered infectivity and reduced decay, but also improvements in diffusional mobility in liquid culture, as well as tissue trafficking [5,10–12]. Cell surface transmission of (HIV-1) and Human T-cell Leukemia virus Type 1 (HTLV-1) have recently received renewed attention because of their contributions to enhancing tissue infectivity [4,10,13–15]. In the case of retrovirus HTLV-1, non-specific particle adsorption that leads to ‘*biofilmlike*’ surface transfer between cells has been reported [13]. HIV-I spreads rapidly with 100-1000x greater efficiency via cell-cell transmission than with cell-free infection [1,16]. Likewise, vesicular stomatitis virus (VSV) has been shown to spread directly between cells [17].

Conditionally replicating HIV-1 derived lentiviral vectors are widely used for the stable transduction of cells, including quiescent stem cell populations [18,19]. Considerable insight has been gained in recent years into the insertional biosafety of γ-retrovirus and lentivirus vector systems [20,21]. Additionally, approaches exist for promoting tissue- and target cell receptor specificity or optimizing lentiviral transduction efficiency through pseudotyping [20,22]. We previously demonstrated the inadvertent transduction of bystander cells by cell bound lentivector particles [23]. In a subsequent study we observed the prolonged cell surface retention and delayed entry of particles prior to entry [24]. However, how the competing fates of cell entry versus dissociation and cell-cell surface transfer of replication deficient retrovirus-derived particles could be reconciled has remained unclear [23,25–27].

Here we show that at limiting input, VSV-G lentivector particles retained on the cell surface transfer with a proportional efficiency to other cells that surpasses cell-free (i.e. neat particle) transduction. We show that cell-cell transmission involves vector stabilization on the cell surface and delayed neutralization kinetics during the transfer to bystander cells. The data suggest that the surface attachment of VSV-G lentivector particles prior to uptake constitutes a potentially important and underexplored interval for transmission of recombinant lentivector particles.

## Materials and Methods

### Vector production

Human 293T kidney fibroblasts cells were seeded at a density of 1.6 x 10^8^ cells per 15-cm tissue culture dish (Corning), precoated with 0.01% poly-L-lysine (Sigma). The lentivirus transfer vector pLVCG was a kind gift from Francesco Galimi. The GFP-Vpr plasmid used in imaging experiments was provided by Eric Barklis (Portland, OR). In select fluorescent experiments, the GFP reporter was replaced by cloning in the fluorescent reporter, mCherry. Calcium phosphate transfection of 4^th^ generation lentivector packaging (pMD-Lg/p-RRE, pRSV-Rev) and VSV-G envelope plasmids was performed in the presence of DMEM (Gibco), 10% FBS (Gibco), 1% Penicillin/ Streptomycin (Pen/Strep, Gibco). Vector supernatant was harvested 36, 48, and 72 hours later, filtered through a 0.45 µm filter and ultra-concentrated over 30 hours at 7300 RCF, and the pellet was resuspended in Iscove’s media (Gibco) and stored at -86°C until use. Limiting dilution titers were determined by FACS and calculated using 293T cells, as previously described [28]. For transduction, cells were washed and resuspended in corresponding media (described below), with 4-8 µg/ml protamine sulfate (MP Biomedicals). Transductions took place at 37°C for specified lengths of time.

### Cell culture

Jurkat cells (a gift from Dr. Brian Druker, obtained from ATCC) and SupT1 cells (human T cell lines received from the NIH AIDS Reagent Program) were grown in RPMI (Gibco) supplemented with 10% FBS (Gibco) and 1% Pen/Strep. Human kidney fibroblasts (HEK) 293T (a gift from Dr. Markus Grompe at OHSU) [29] and NIH 3T3 murine fibroblasts were grown in DMEM (Gibco) supplemented with 10% FBS and 1% Pen/Strep. Experiments involving fibronectin fragment CH296 (Takara Mirus, Madison, WI) used a final concentration of 5 µg/ml, unless otherwise noted.

### VSV-G neutralization

The VSV-G neutralizing antibody was harvested from supernatant of hybridoma CRL-2700 (American Type Culture Collection), and concentrated with Amicon Ultra columns (Millipore). Neutralizing activity was assayed by adding serial dilutions of anti-VSV-G antibody to 1x10^5^ vector-exposed Jurkat cells overnight at 37°C. The concentration needed to effectively neutralize 100% of vector particles was determined 48-72 hours later by absence of GFP fluorescence via flow cytometry.

### Enzyme-linked immunosorbent assay p24 (ELISA)

To determine physical vector titers (ng/mL) a commercially available ELISA kit was used (Perkin-Elmer, Boston, MA). To assure linear range absorbance measurements, samples of cell-free and cell-bound vector were serially diluted. Control samples were run in triplicate and a standard curve was generated according to manufacturer’s protocol.

### Flow-cytometry

Retroviral transduction was analyzed by GFP expression using a FACS-Calibur instrument (BD Biosciences) or MACSQuant Analyzer (Miltenyi Biotech), and Flow Jo software (Tree Star, Ashland, OR). At least ten thousand events per sample were collected for any given experiment. Cells of hematopoietic lineage were determined by staining for CD45 (human, APC conjugated, Biolegend). Non-viable cells were excluded from analysis by uptake of propidium iodide (1 µg/ml solution).

### Fluorescent microscopy

Cells were prepared by incubation in Hoechst 33342-containing media (5 mg/ml) for 45 minutes to stain nuclei (live cells) or staining with 4’,6-diamidino-2-Phenylindole Dihydrochloride (DAPI) for 1 minute (fixed cells). Transductions were performed with GFP*vpr* vector in the presence of 8 µg/ml protamine sulfate for 1 hour at 4°C. Cells were then washed, fixed with 4% paraformaldehyde, and washed with PBS. To stain the actin cytoskeleton, Alexa Fluor 555 phalloidin (Life Technologies), was added to fixed cells for 30 minutes, then washed off with PBS. Transduced cells were stained with anti-VSV-G antibody (Sigma), followed by staining with anti-rabbit Alexa Fluor 647 (Life Technologies). Slides were mounted with fluoromount G (SouthernBiotech). Images were acquired using a Nikon epifluorescent inverted microscope with a SPOT-2 digital CCD camera (Diagnostic Instruments), or a Zeiss epifluorescent inverted microscope (Carl Zeiss MicroImaging, Inc.) with an ORCA-ER CCD camera (Hamamatsu Corporation). Separate layers for brightfield and fluorescence were captured and imported to generate overlay images using SoftWoRx Explorer (Applied Precision LLC, Issaquah, WA). Any adjustments in contrast or intensity were carried out using Photoshop software (Adobe Systems Inc., Seattle WA). Any adjustments were applied to the whole image.

### Transmission Electron Microscopy

Jurkats (1x10^6^) were transduced overnight at 37°C (MOI 25). The following morning, cells were washed twice with PBS + 2% FBS, then resuspended in Karnovsky fixative (100 mM sodium cacodylate, pH 7.2, 2.5% glutaraldehyde, 1.6% paraformaldehyde, 0.064% picric acid, 0.1% ruthenium red) for 1 hour on ice. Fix was then removed, cells were thoroughly rinsed in water, dehydrated, infiltrated overnight in 1:1 acetone: Epon 812, infiltrated 1 h with 100% Epon 812 resin, and embedded in resin. After polymerization, 60- to 80-nm thin sections were cut on a Reichert ultramicrotome, stained 5 min in lead citrate, rinsed, post-stained 30 min in uranyl acetate, rinsed, and dried. EM was performed at 60 kV on a Phillips Morgagne TEM, equipped with a CCD, and images were collected at original magnifications of 1,000 - 37,000x.

### Statistics

Statistical significance was determined by performing a paired 2-tailed Student’s *t*-test. Tests are appropriately labeled whether equal or unequal variance was assumed. P values less than 0.05 were considered statistically significant.

## Results

### Efficient cell-cell transfer of vector particles

**Figure 1:**
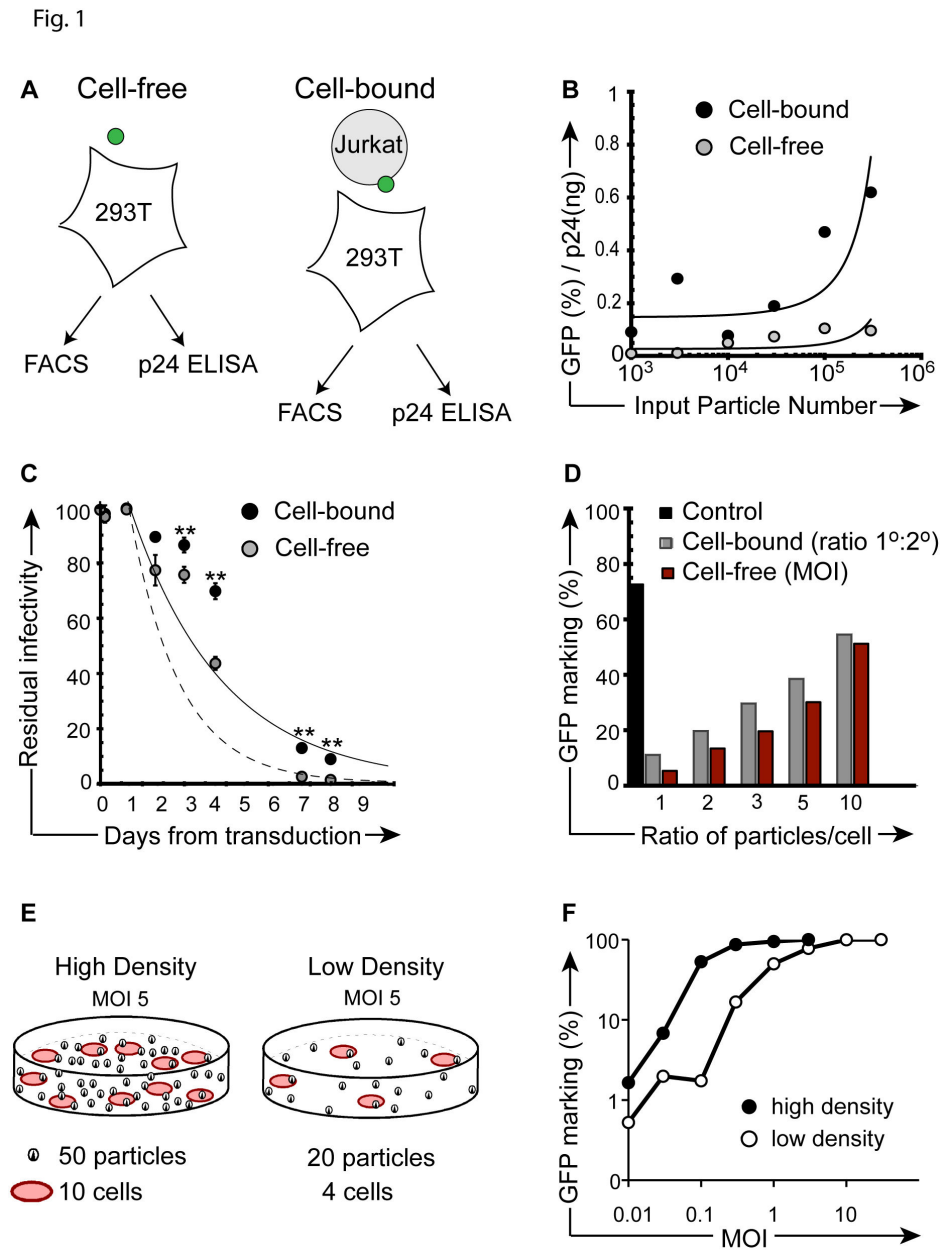
Enhanced gene transfer after direct cell-cell contact of 1^o^ and 2^o^ targets. (**A**) Experimental design, illustrating cell-free (left) and cell-bound (right) transduction strategies. (**B**) Ratio of relative infectivity (% GFP / ng p24) after 24-hour direct cell-free transduction versus transduction by (Jurkat) cell-bound particles following co-culture. The *y*-axis ratio reflects GFP marking (FACS) per ng input vector (p24 ELISA) at increasing vector particle numbers (*x*-axis), corresponding to MOI 0.01-3. Refer to *Methods* section for detailed procedure. (**C**) Residual infectivity of cell surface bound versus cell-free particles. Particles were kept at 37^o^C and identical aliquots (MOI 1, based on vector titer) were removed at indicated time points for transduction culture of 1 x10^5^ 293T cells (cell free, black circles). For the cell-bound conditions, the same number of vector particles were arrested on the surface of 1^o^ target Jurkat cells (MOI 1) at 4^o^C, before shift to 37^o^C. Aliquots of 1 x10^5^ cells were subsequently placed in coculture with 293T 2^o^ targets at the indicated time-points. A 2-tailed paired Student’s *t* test was performed; p values ≤ 0.01 are indicated by double asterisks. (**D**) Relative GFP marking in secondary 293T cells after direct co-culture (24 hours) by escalating the number of GFP vector exposed Jurkat cells (1^o^, CD45^+^) to 293T (2^o^, CD45^-^) cells at stable MOI of 1 (*ratio*, gray bars), or by escalating MOI during exposure of primary cells, with matched 1:1 cell numbers (*MOI*, red bars). Direct transduction of 293T cells with cell-free vector (black bar, control). (**E**) Illustration of experimental design used in (F). (**F**) Transduction of Jurkat cells under high cell density transduction (1x10^6^ cells per ml, black circles) or low cell density (1x10^5^ cells per ml, open circles) transduction conditions over a range of MOI. Cells were transduced in the presence of 4 µg/ml protamine sulfate. Following a 3-hour transduction at 37°C, cells were washed, placed back in culture at 37°C, and flow cytometry was performed 72 hours later. All experiments were repeated with similar results.

Both HIV-1 and vesicular stomatitis virus (VSV) spread efficiently between cells via cell-cell transmission [1,16,17]. While *ex vivo* transduction using VSV-G pseudotyped HIV-derived particles does not involve their replication, or membrane budding and spread, we hypothesized that the prolonged surface retention we recently described may account for particle transmission via direct cell-cell transfer [23,24]. To test the relative transduction efficiency by cell-bound particles, we standardized the amount of input p24(Gag) in cell-free, or cell-bound systems [30,31]. Specifically, we transduced 293T cells with cell-free (i.e. neat) vector particles or through co-culture with cell-associated particles (attached to the surface of Jurkat cells after a 1-hour vector exposure and saline wash) using matched vector particle input (TU/ml) [32]. Gene marking in 293T cells was then determined in both cell-free and cell-bound experimental arms by flow-cytometric (FACS) analysis (Figure 1A). In identically prepared aliquots, the amount of input vector was determined in both experimental arms (cell-free *versus* cell-bound) by p24(gag) ELISA. This allowed us to calculate and compare the relative infectivity (% GFP^+^) per nanogram p24 of particle input. Results demonstrate that the rapid and proportional increase in p24 content in the cell-free experimental arm is not matched by comparable gains in GFP expression. The results at limiting particle input reveal that the relative infectivity (% GFP^+^/ ng p24) favors cell-bound over cell-free vector particle delivery for efficiency (Figure 1B). It is important to note that a fraction of vector particles is lost in the saline wash after the initial transduction culture and therefore not available for transduction of 293T cells, implying a potential underestimate of efficiency for secondary transduction events by cell-bound particles. Timed transduction experiments to determine if cell surface bound particles are subject to an altered half-life, shown for HIV-1 [5], demonstrated statistically significant differences in the half-life of cell-bound particles 3 days following the initial vector exposure (Figure 1C). These results suggest that particles are stabilized on the cell surface [33]. To corroborate the increased efficiency of direct cell-cell transfer at limiting particle input, we hypothesized that increases in cellular carriers should lead to greater gains in transduction than simple escalation of MOI. Keeping the total number of particles available for 2^o^ transduction constant, one arm used escalating particle numbers in Jurkat cells, while the other arm employed increasing vector-exposed Jurkat cell numbers during coculture (increasing 1^o^ to 2^o^ cell ratio). For example, Jurkats were exposed to vector particles at MOI 3 and placed in coculture with 293T cells at a 1: 1 cell ratio; whereas in the arm escalating 1^o^ ‘carrier’ cells, Jurkats were transduced at MOI 1 and put in coculture with 293T cells at a cell ratio of 3:1. Results showed that changes in the ratio of 1^o^ to 2^o^ target cell resulted in 1.5 to 2-fold greater gains in gene transfer to 2^o^ targets than merely increasing the number of particles (i.e. MOI) during 1^o^ vector exposure (Figure 1D). Given the relatively shorter exposure here (i.e. 24 hours), extended half-life of cell-bound vector particles would unlikely explain the observed differences, whereas the experiments suggest that cell density itself may increase transduction efficiency independent of particle concentration, or multiplicity of infection. Indeed, we observed 3 to 30-fold increases in transduction when cells were cultured under high density (10x) versus low-density culture conditions (Fig 1EF). In total, these data reveal the comparative efficiency of direct lentivector particle transmission between cells.

### Vector particles do not form surface aggregates

**Figure 2:**
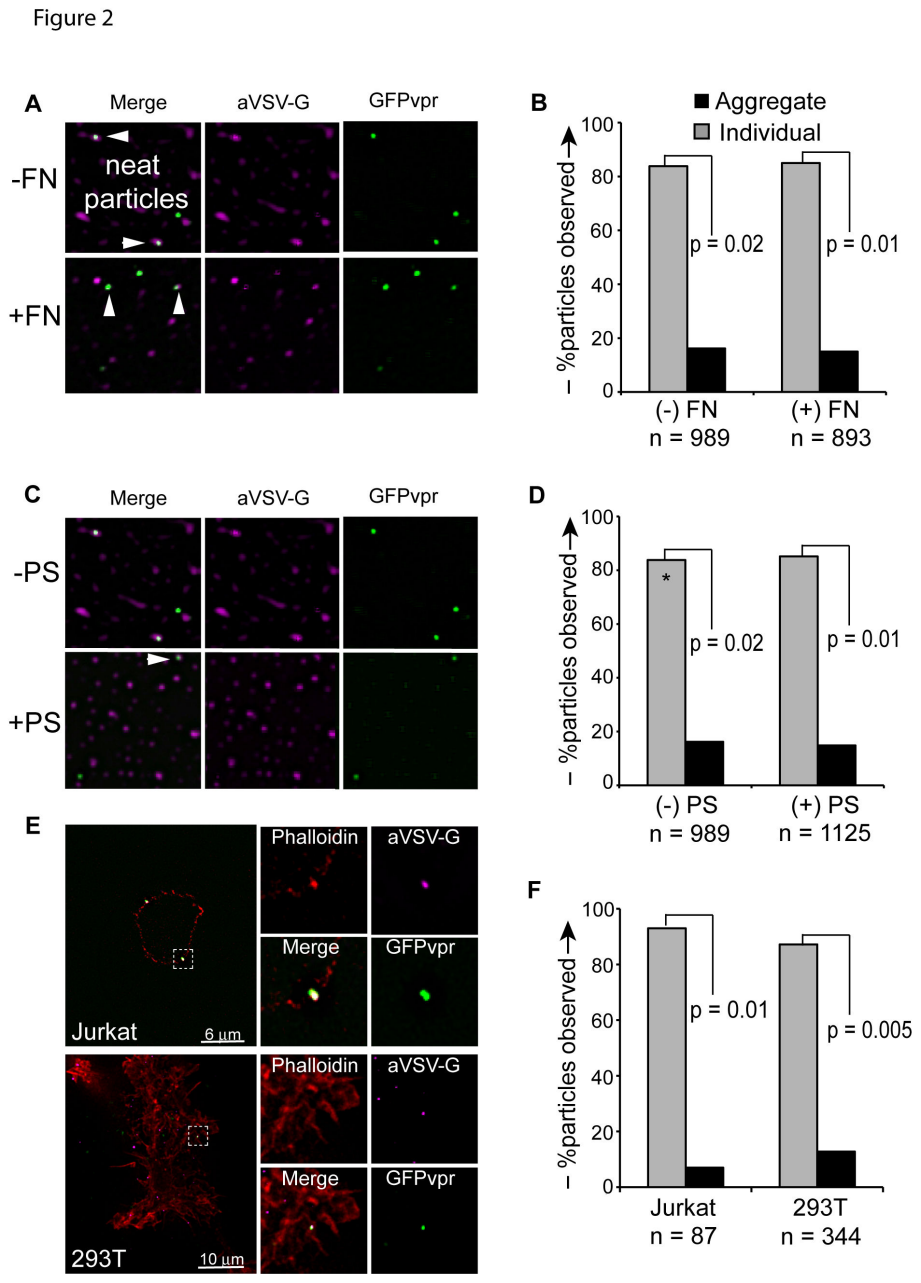
VSV-G pseudotyped lentivector particles do not form aggregates. (**A**) GFP*vpr* labeled vector particles were allowed to settle on glass slides in the absence (top) or presence (bottom) of recombinant fibronectin fragment, CH296, stained with anti-VSV-G antibody and Alexa Fluor 647 anti-mouse secondary antibody, and visualized via deconvolution microscopy. (**B**) Particles either counted as “individual” (gray bars) or as “aggregate” (black bars) if 2 or more individual particles were observed to occupy contiguous space. Significance was determined by performing a Student’s 2-tailed *t-test*, assuming unequal variance between groups. (**C**) GFP*vpr* labeled particles were allowed to settle on glass slides in the absence (top) or presence (bottom) of 8 µg/ml protamine sulfate. (**D**) Particles either counted as “individual” (gray bars) or as “aggregate” (black bars) if 2 or more individual particles were observed to occupy contiguous space. Asterisk indicates that data set for “(-PS) control is the same from (B), “(-FN)” control. Significance was determined by performing a Student’s 2-tailed *t-test*, assuming unequal variance between groups. (**E**) Jurkat (top panels) and 293T cells (bottom panels) were exposed to GFP*vpr* vector particles (green) for 1 hour at 37C, washed, and fixed with 4% paraformaldehyde. Cells were stained with phalloidin (red) and anti-VSV-G antibody (magenta), followed by staining with anti-rabbit Alexa Fluor 647 (blue), and imaging via deconvolution microscopy. (**F**) Particles either counted as “individual” (gray bars) or as “aggregate” (black bars) if 2 or more individual particles were observed to occupy contiguous space. Significance was determined by performing a Student’s 2-tailed *t-test*, assuming unequal variance between groups.

HTLV-I particles are transmitted between cells in highly infectious, biofilm-like, surface aggregates that are rich in extracellular matrix proteins [13,34,35]. Therefore, we investigated the possibility that cell-bound transfer of lentivector particles may be enhanced and half-life may be extended via formation and cell-cell transmission of surface aggregates. GFPvpr-labeled particles were visualized via deconvolution microscopy. GFPvpr fusion protein associates with gag protein in the mature virion, and allows for fluorescent detection of individual particles [36,37]. To control for any aggregation of neat vector, GFPvpr-labeled particles, resuspended and cryopreserved in Iscoves media, were thawed and observed to settle on glass slides as individual, dispersed particles. Neat particles did also not form aggregates in the presence of protamine sulfate, or recombinant extracellular matrix fibronectin protein fragment, CH296, frequently used to enhance transduction by colocalizing particles to non-adherent hematopoietic target cells (Figure 2A–D). Likewise, when Jurkat or Sup-T1 (both lymphoid), or 293T (fibroblast) cells were exposed to vector for 1 hour, followed by a PBS wash, only rare particle surface aggregates were observed (Fig. 2EF). Others have shown that the efficiency of GFPvpr tag incorporation varies and we therefore validated the lack of surface aggregation using anti VSV-G antibody [38]. These experiments show that cell-free particles are not inherently prone to aggregation. Similarly, their attachment to cells does not promote the formation of multi-particle aggregates to account for the observed efficiency of cell-bound particle transmission.

### Cell-bound particles escape neutralization

**Figure 3:**
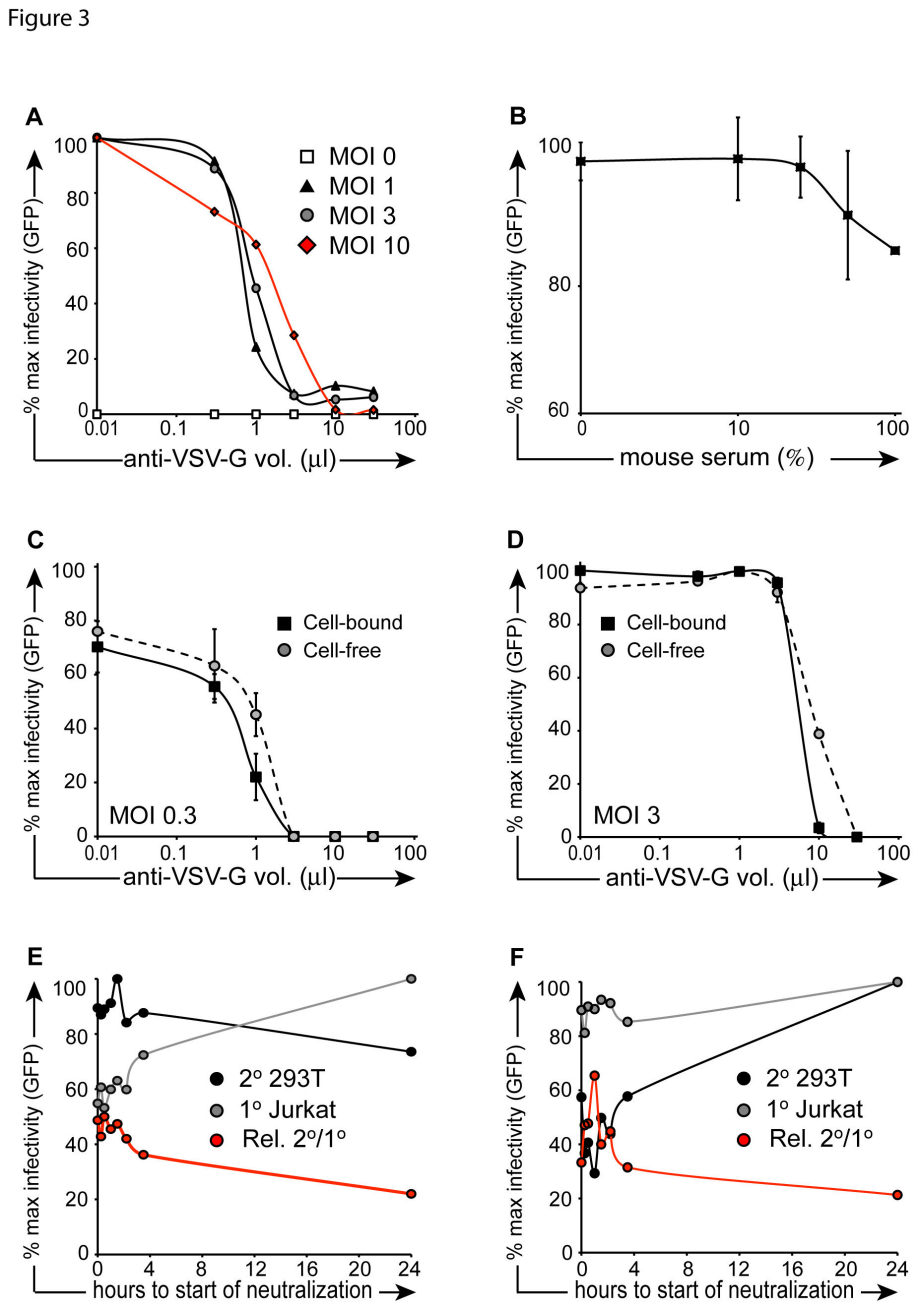
Neutralization kinetics of cell-free vector. (**A**) Vector particles were incubated in escalating concentrations of anti-VSV-G neutralizing antibody for 1 hour at 4°C in the presence of 4 µg/ml protamine sulfate. Particles were then placed on 293T cells for overnight transduction at 37°C. Cells were washed after 24 hours, and FACS was performed 48 hours later. (**B**) Experiment was performed as described in (A) with the exception that mouse serum was used instead of VSV-G neutralizing antibody and the neutralizing incubation was performed at 37^°^C for the purpose of minimizing cell death resulting from serum incubation (CD). In the cell-bound condition (black squares), 1x10^5^ prechilled Jurkat cells were exposed to vector (**C**: MOI 0.3; **D**: MOI 3) for 1 hour at 4°C in the presence of 4 µg/ml protamine sulfate, then placed in co-culture with 2.5x10^4^ pre-plated 293T cells at 37^°^C. Cell-free samples (gray circles) were prepared in parallel, in the absence of Jurkat cells. Escalating concentrations of anti-VSV-G neutralizing antibody were added to co-cultures and incubataed overnight at 37°C. Cells were then washed, and FACS was performed 48 hours later (EF). 1x10^5^ prechilled Jurkat cells were exposed to vector (MOI 3) for 1 hour at 4°C, then placed in co-culture with 2.5x10^4^ pre-plated 293T cells at 37°C. At serial time points (*x*-axis), 0.5% anti-VSV-G neutralizing antibody (E) or 10% mouse serum (F) was added to the transduction culture. Target 293T cells were washed 24 hours after vector exposure, and FACS was performed 48 hours later. CD45-APC antibody was used to exclude primary Jurkat cells (gray circles) from secondary target 293T cells (black circles). Red line depicts % 293T GFP+ events/ % Jurkat GFP+ events.

We and others have shown that VSV-G pseudotyped vector is inactivated by serum complement as well as neutralizing antibody [9,37,39]. We confirmed the efficiency of cell free vector inactivation with neutralizing antibody, by testing residual infectivity in 293T fibroblasts. Vector inactivation (1 hour) followed a dose–response trend in which increasing MOI required a larger volume of anti-VSV-G antibody for deactivation. Nevertheless, 30 µl of anti-VSV-G antibody were found to inactivate the majority of particles at all MOIs tested (Figure 3A). Predictably, concentrated VSV-G antibody neutralized particles more effectively during a 1-hour incubation (MOI 1) than complement containing mouse serum (Figure 3B).

Having shown the relative efficiency of inactivating cell-free particles, we considered that cell-bound particles might be protected from neutralization following attachment without an implicit commitment to cellular uptake. We first determined endpoint neutralization for particles attached to the cell surface at 24hrs. Jurkat cells were exposed to vector then placed in co-culture with secondary target 293T cells. Contrary to our hypothesis, when measuring endpoint neutralization after 24 hours, cell-bound particles were slightly more sensitive to antibody than cell-free particles (Fig. 3CD). This suggests that particles bound to cells are not inherently protected from inactivation during transfer between cells in co- culture. We next examined the kinetics of inactivation of cell-bound vector during transmission to secondary targets. Jurkat cells were exposed to vector particles in the cold (4^o^C) and placed in co-culture with pre-plated 293T cells. At serial time points from 0 to 24 hours, anti-VSV-G neutralizing antibody or mouse serum were added to co-cultures. We observed an expected decrease in primary cell transduction while the percentage of transfer to secondary (target) cells remained largely unchanged. Thus, the relative efficiency of secondary transfer was 2-fold higher at earlier time points (0 to 4 hours) than at later time points (Figure 3E) At time points approaching 24 hours, the efficiency of secondary transfer decreased, eventually matching the efficiency of transfer in media-treated controls after 24 hours (not shown). We repeated the experiment in the presence of mouse serum and observed similar results (Figure 3F). In sum, fewer cell-bound particles are inactivated at early time points and particles attached to the cell surface following primary transduction culture transfer efficiently to secondary targets escaping neutralization.

### Particle transduction and neutralization depend on the availability of surface binding sites

**Figure 4:**
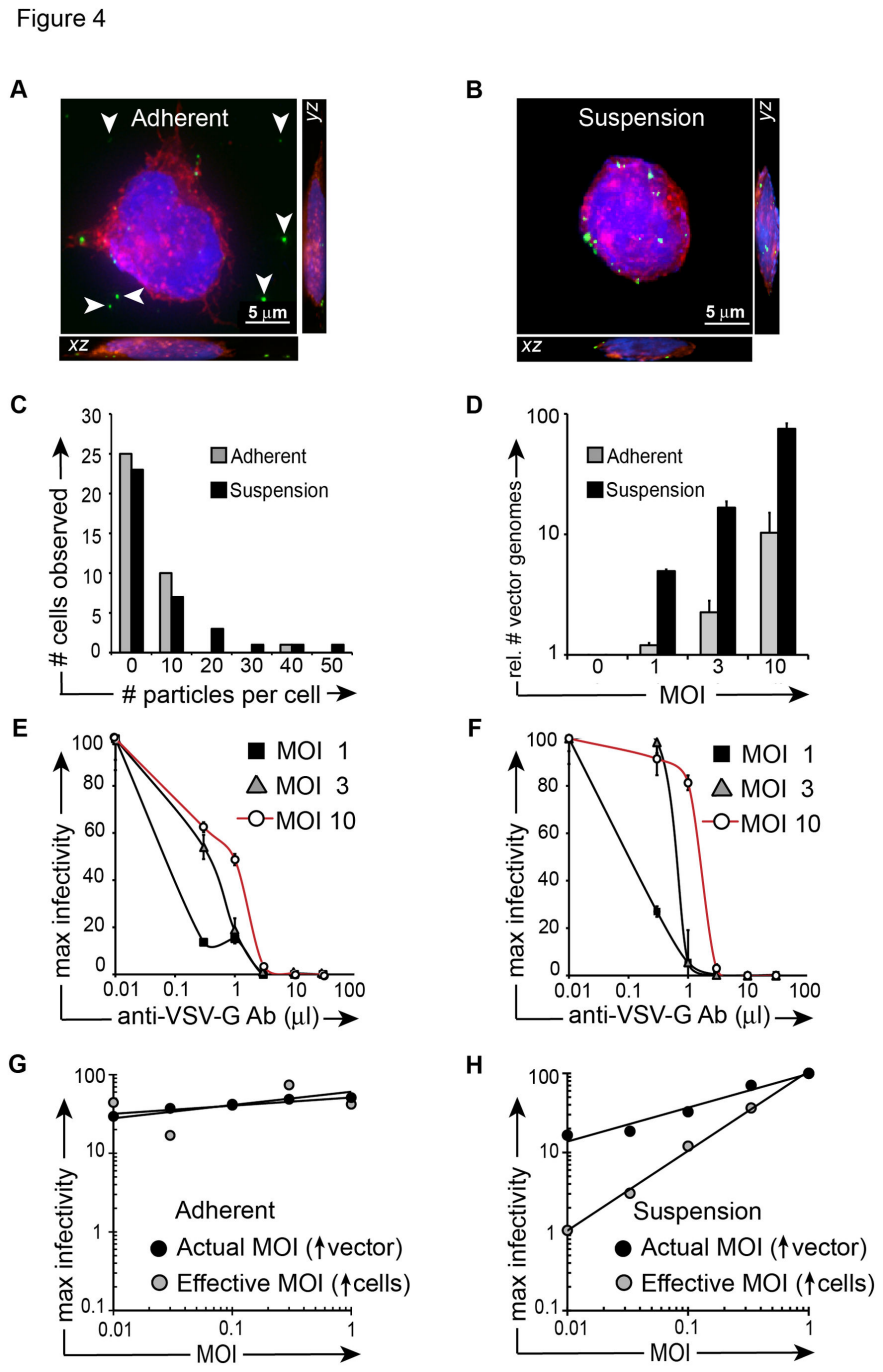
Neutralization is dependent upon cell mobility. (**A**) Merged *z*-stack of transduced adherent cell shown alongside *yz* and *xz* planes. 6.0x10^4^ NIH 3T3 cells were allowed to adhere to a chambered well for 3 hours. Cells were then transduced for 30 minutes at 4°C, fixed with 4% paraformaldehyde, washed with PBS, permeabilized with NET buffer, stained with Alexa Fluor phalloidin 555 (red), and DAPI (blue). (**B**) Merged *z*-stack of transduced suspension cell shown alongside *yz* and *xz* planes. 6.0x10^4^ NIH 3T3 cells were prepared as described in (A) with the exception that cells were transduced following 3 hours of suspension culture in polystyrene tubes instead of plating in adherence to chambered wells. Once cells were fixed and stained, they were placed in chambered wells for microscopy. Scale bars in (A), (B) are 5 µm. (**C**) The experiment described in (A) and (B) was performed twice and the average number of particles per cell was determined by counting particles in 36 cells per cohort. The frequency of cells observed to have a specific number of particles bound to the cell surface was calculated using Microsoft Excel. (**D**) NIH3T3 cells were exposed to increasing concentrations of vector as in (C), cells were immediately fixed, then cDNA was synthesized, and quantitative RT-PCR was performed with primers that amplified GFP (vector) or GAPDH. The relative quantitation of GFP (i.e. vector genomes) is shown on the *y*-axis. (**E**) 293T cells were allowed to adhere to tissue culture-treated plates for at least 4 hours (“Adherent”, left), or were left in suspension (**F**) in polypropylene tubes (“Suspension”, right). Cells were transferred to 4C for 1 hour, followed by a 1-hour vector exposure (MOI 0, 1, 3, 10). Following vector exposure, cells were transferred to 37^o^C and serial concentrations of anti-VSV-G neutralizing antibody were added to the media. FACS was performed 72 hours later. (**G**) Vector was added to pre-plated 293T cells in serially increasing concentrations while media volume and cell number remained constant (actual MOI, black circles). Increasing numbers of 293T cells were plated on 12 well poly-L-lysine-coated plates, while keeping vector concentration and media volume constant (effective MOI, gray circles). Cells were washed after 4-hour vector exposure in the presence of 4 µg/ml protamine sulfate. FACS was performed 72 hours later. (**H**) The experiment was performed as described in (G) with the exception that 293T cells were kept in suspension during 3-hour transduction in polystyrene tubes. Cells were then washed and transferred to 12-well plates. FACS was performed 72 hours later.

Therefore, we next examined vector binding- and neutralization properties in adherent versus non-adherent fibroblast cells. To determine if more vector binding sites are available when cells are in suspension versus adherence, we plated NIH3T3 murine fibroblasts in polystyrene tubes (suspension) or on TC-treated plates (adherence). These cells, rather than (smaller) 293T cells, were chosen to maximize binding sites for the study of vector-cell surface binding in adherence as well as suspension [40]. We exposed cells at 4^o^C for 30 minutes to GFPvpr tagged lentivector, then immediately fixed the cells to avoid transduction and proviral GFP expression. We performed fluorescent deconvolution microscopy on vector-exposed cells and observed an average of 2 particles bound per adherent cell and 6 particles bound per suspension cell (Fig. 4AB) (not statistically significant). We examined the frequency of particles observed per cell and did not detect a difference between particles bound in adherent versus suspension conditions (Figure 4C). We next performed qRT-PCR to quantify vector genomes bound to adherent versus suspension NIH3T3 cells, harvested immediately after a 30-minute GFPvpr vector exposure, and we observed consistent and increasing gains in vector genomes bound to suspension cells at MOI >1, when compared to adherent cells (Figure 4D). Finally, we examined vector neutralization characteristics when 293T cells were cultured under adherence or kept in suspension, then exposed to vector at escalating MOI, followed by incubation with anti-VSV-G neutralizing antibody. Consistent with RT-PCR results for vector genomes, we observed that cells transduced at MOI 3 or 10 under suspension conditions exhibited 30% higher residual infectivity following incubation with anti-VSVG antibody than cells transduced in adherence (Fig. 4EF).

In Figure 1F we showed that increasing cell density positively impacts transduction. Next we tested the effect of cell density on transduction of 293T cells cultured in adherence or suspension. Under otherwise identical conditions, adherent 293T cells were pre-plated and transduced on poly-L-lysine-coated plates, while 293T cells in suspension were transduced in polystyrene tubes. Results show that transduction with increased cell density did not lead to gains in infectivity when cells were transduced under adherent conditions (Figure 4G). In contrast, substantial gains in infectivity were observed as cell density increased for cells transduced under suspension conditions (Figure 4H). These results suggest that cell surface binding site availability and mobility during suspension culture are responsible for enhanced the transduction efficiency in suspension and cell-cell transfer.

Finally, to determine whether primary cells were involved in active signaling that influenced cell-cell transfer, we conducted experiments to test the efficiency of direct cell-cell transfer of lentiviral vector particles following chemical fixation of primary cells. Jurkat cells were fixed with 4% paraformaldehyde, followed by vector exposure and co-culture with live 293T cells. At a constant vector dose (MOI 1) no significant secondary transduction was observed. We repeated the experiment across a range of MOI (0.5-10) and observed similar results (no secondary transduction, Figure S1A). These results suggest that primary cells are involved in active signaling to secondary cells prior to or during cell-cell transfer of vector particles.

### Visualizing particle surface transfer between cells

**Figure 5:**
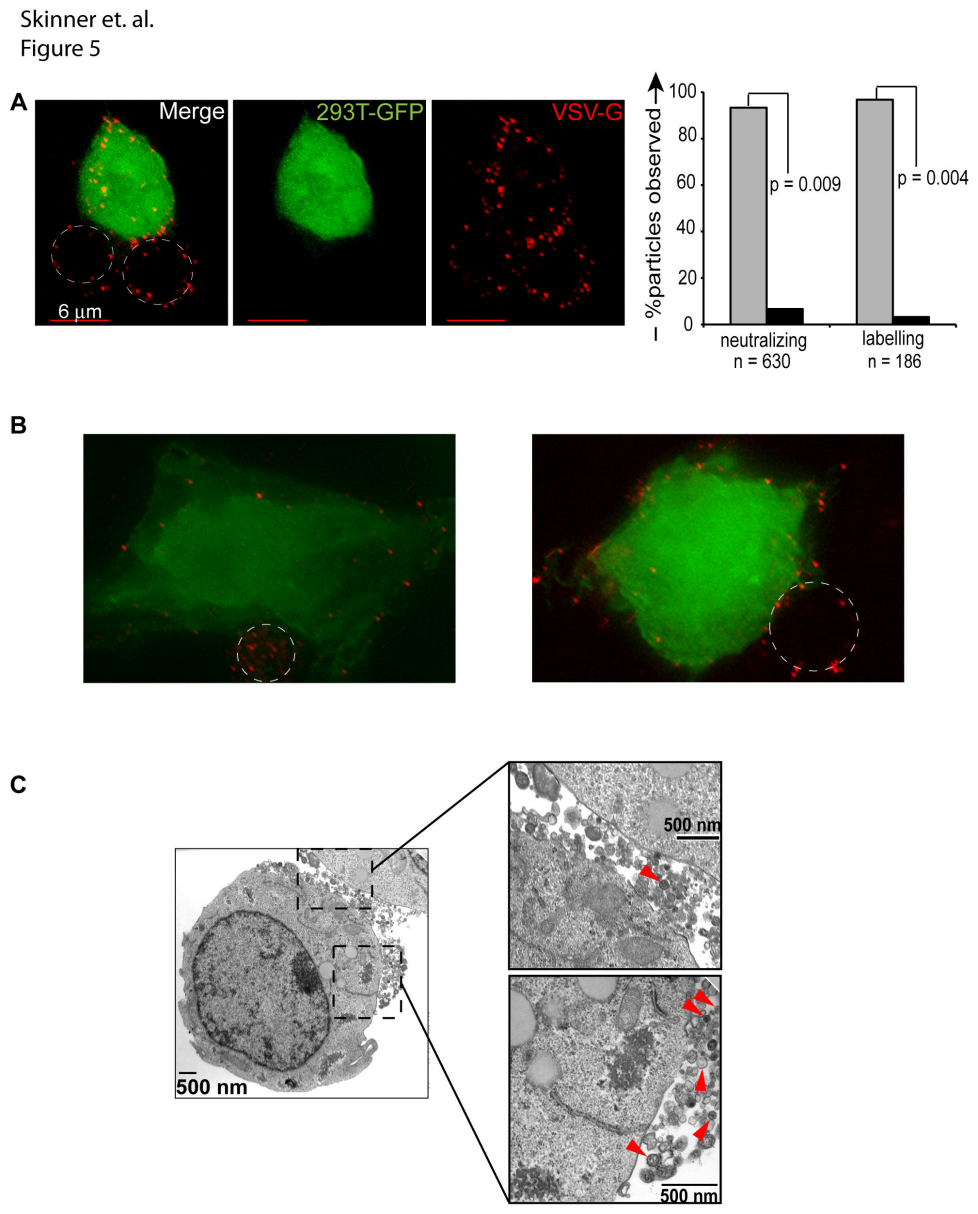
Vector particles do not consistently polarize to areas of cell-cell contact. (**A**) Jurkat cells were exposed to mCherry*vpr* vector (red) for 1 hour at 4°C, cells were washed and placed in co-culture with GFP-expressing 293T cells (green) that were pre-plated on glass cover slips. Cells were washed with PBS, stained with anti-VSV-G antibody (shown) or neutralizing anti-VSV-G antibody (not shown), fixed with 4% paraformaldehyde, and then stained with Alexa Fluor 647 anti-rabbit secondary antibody (magenta). The percentage of single or aggregated particles was enumerated in cells treated with neutralizing versus labeling VSV-G antibody. Statistical significance was determined via a 2-tailed Students *t* test assuming unequal variance. (**B**) A representative image was acquired as described in (A) and used to generate a projection image of 13 *z*-stacks in softWoRx Explorer. (**C**) 1x10^6^ Jurkat cells were transduced overnight at 37°C (MOI 25). Cells were washed twice in the morning, pelleted, fixed for 1 hour in Karnovsky’s fixative, and prepared for electron microscopy. Panel on left is magnified 14,000x; the right top panel is magnified 36,000x. Red arrowheads highlight vector particles.

To examine the transfer of cell-bound particles more closely we performed fluorescent deconvolution microscopy during cell-cell vector transfer. Vector particles labeled with mCherry were bound to the surface of Jurkat cells for 1 hour at 4°C in the presence of VSV-G neutralizing antibody, cells were washed, then placed in co-culture for 1 hour with GFP-expressing 293T cells. During this brief co-culture, particles were transferred from the surface of the primary vector-exposed cell, to 293T cells (Figure 5A). To determine whether neutralization-resistant particles are transferred to secondary targets as single particles or in aggregates, we tallied the numbers of each and found that the majority (97%) were single particles transferred in the presence of neutralizing VSV-G antibody, while only a small number were aggregates (Figure 5A, right). Contrary to reports that virus particles preferentially polarize to areas of cell – cell contact, we did not consistently observe this phenomenon in our fluorescent microscopy studies, as ascertained by generating merged *z*-stacks of representative images (Figure 5B) [41]. To validate the results further, we repeated the experiment using a different (labeling, i.e. non-neutralizing) VSV-G antibody. Finally, when we performed limited transmission electron microscopy on vector-exposed Jurkat cells, we again observed that a majority of particles at the cell surface were single particles, while a smaller number were aggregates, illustrated in (Figure 5C). These experiments indicate that protection from VSV-G neutralizing antibody is not dependent on specific neutralization of multi-particle assemblies.

## Discussion

We previously reported that the prolonged surface adherence of HIV-1 derived lentivector to hematopoietic cells results in inadvertent 2^o^ transduction events [23]. We have also shown that particle transmission to 2^o^ targets correlates inversely with the cell-specific rate of vector clearance from the surface of the 1^o^ target [24]. Our observations are not limited to species-specific or cell-intrinsic phenomena, as we have obtained similar results of efficient cell-cell transduction at low MOI utilizing multiple donor cell > target cell strategies in this and previous works: human myeloid K562 > 293T, 293T > Jurkat, human SupT1 > 293T, human lymphoid Raji > 293T, human hepatoma HepG2 > 293T, murine L1210 > murine 3T3, murine whole bone marrow > 3T3, murine lineage depleted whole bone marrow > murine 3T3 [24,37]. Together, our observations seemed to point to a critical role for the initial attachment by pseudotyped vector particles to cells and their subsequent delayed clearance, either through cell entry or via transmission to bystander targets. For the first time, we now show that VSV-G pseudotyped replication-deficient lentivector particles are protected from neutralization and transduce cells more efficiently via a cell-bound rather than cell-free mechanism.

The comparison of (conventional) cell-free transduction by non-replicating vector particles with direct cell-cell transmission (cell-bound mode) presents a challenge in terms of experimental design, as cell-bound particles continuously enter the primary (non-target) cells, with a decreasing proportion remaining on the surface and amenable to transmission and inactivation. Initially, we therefore sought to correlate input vector based on viral gag protein (p24) with the proportion (%) of GFP expressing target cells in bulk culture, across a range of MOI. These studies establish for the first time the surprising efficiency of surface attachment and cell-cell spread of pseudotyped vector particles among cells, long known for replicating HIV-1 and HTLV-1 [1,15]. This observation would seem especially relevant at limiting vector particle input, most likely encountered during inadvertent transmission of residual particles following transplantation of *ex vivo* transduced and saline-washed cells [7,23].

We showed that, mechanistically, cell-cell transduction efficiency correlates with increased half-life (Figure 1C) and increased mobility (Figure 1D, Figure 4H). It is tempting to speculate that increased mobility in the co-culture system results from effectual concentration of vector particles as cell-free (i.e. media) volume during co-culture is theoretically less than cell-free volume during primary transduction. To address this issue, we performed mathematical calculations for cell-free and cell-cell culture conditions and determined that the media volume in both conditions was near-equivalent (Figure S1B). Hence, any increased mobility we observed under co-culture conditions cannot be attributed to concentration of vector particles. We further demonstrated that cell-cell transduction efficiency correlates with cell density and increased abundance of surface binding sites on non-adherent cells (Figure 4F). Moreover, we demonstrated that cell-cell transfer of vector particles was abrogated when primary cells were fixed, suggesting that primary cells are involved in active signaling to secondary cells during, or prior to, cell-cell transfer. These results are broadly consistent with studies showing that productive HIV-1 transfer is substantially enhanced under culture conditions that permit cell-cell contact in liquid phase [2,41,42]. In distinction to replicating virus, we found no gains over time in the residual infectivity of cell-associated particles (Fig. 4AB) [5,10].

Contrary to HTLV-1 particle transmission in complex biofilm structures with extracellular matrix proteins [13], we did not observe cell-free particle aggregates. Aggregation was also not seen with commonly used polycations or recombinant fibronectin frequently used to enhance vector transduction. Nevertheless, in analogy to the increased transmission efficiency of HIV-1 and the spread of replication deficient MLV particles in producer cell cultures we observed the direct surface transmission of individual, replication-deficient lentivector particles upon cell-cell contact (Figure 5) [14,43]. Our findings echo the cell-cell transmission of γ-retrovirus and HTLV-1, but argue that neither active viral replication, nor polarized budding are required for directional transmission of surface bound particles [13,14,44].

**Figure 6:**
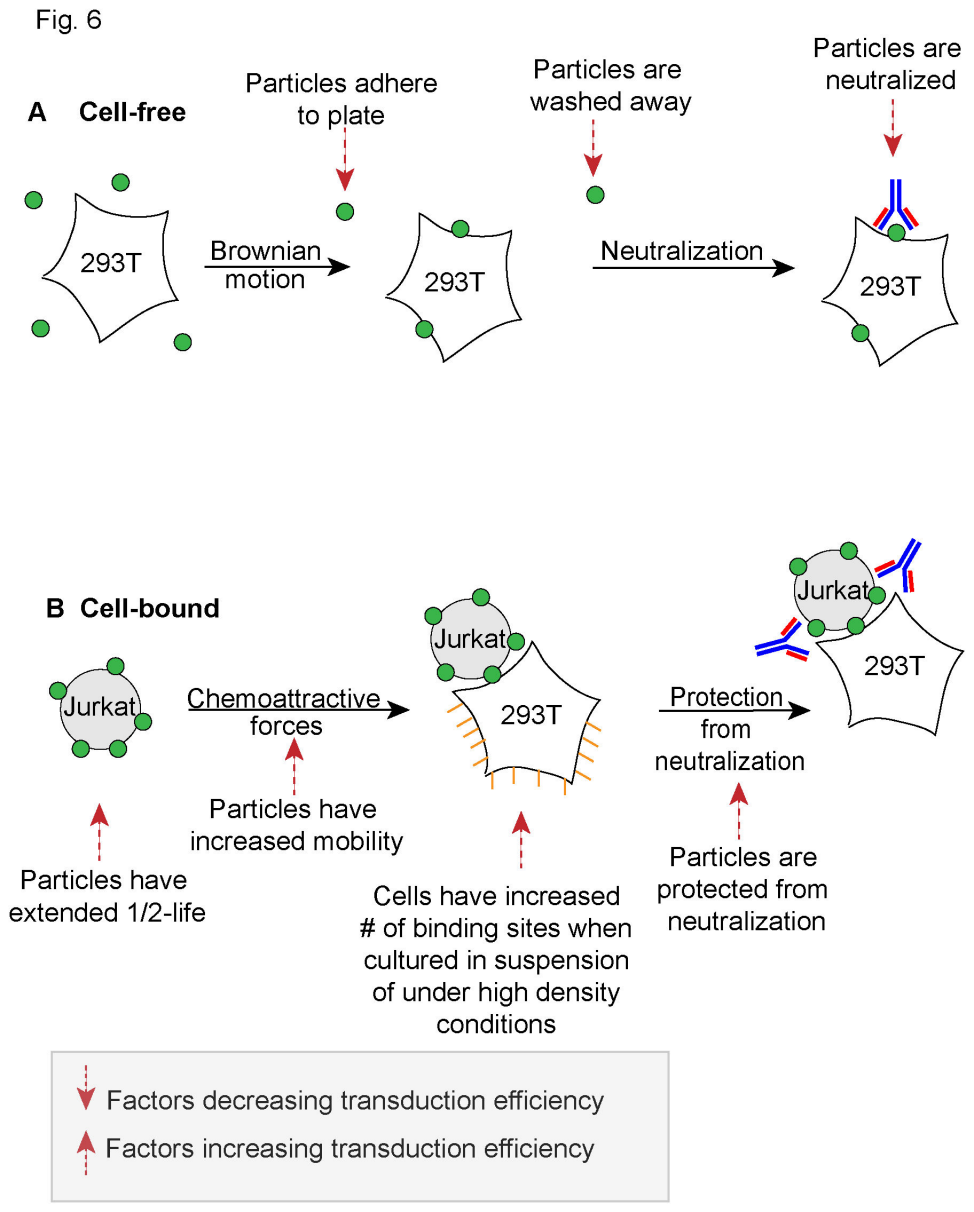
Lentivector cell-cell transmission. (**A**) Cell-free transduction. Several factors are shown that negatively impact transduction efficiency. (**B**) Cell-bound transduction. Several factors are shown that improve transduction efficiency.

In aggregate, our observations suggest that several factors govern the cell-cell transmission of replication deficient, pseudotyped vector particles (Figure 6). These factors include increased half-life, gains in mobility during suspension culture, increased availability of cell surface binding sites under specific (high density and/or suspension) conditions, and delayed neutralization of surface-bound particles. In close analogy to work performed with HIV-1 in the Mothes laboratory, we observed that cell-cell transmission is largely enhanced at low MOI [41,42]. Even as vector particles do not appear to form complex biofilm-like surface aggregates, cell-cell transmission of particles occurs with surprising dilutional efficiency.

## Supporting Information

Figure S1 Supplemental Information. (A) Jurkat cells (1.2x10^6^) were fixed with 4% paraformaldehyde, followed by a 1-hour vector exposure at 4°C in the presence of 8 µg/ml protamine sulfate. Cells were then placed into overnight co-culture with live pre-plated 293T cells (1x10^5^). FACS was performed 48-72 hours later. Secondary transduction was determined in CD45-APC negative GFP+ cells. (B) Mathematical calculations for cell-free and cell-cell culture conditions are shown.
(TIF)

## References

[1] Sato H, Orenstein J, Dimitrov D, Martin M (1992) Cell-to-cell spread of HIV-1 occurs within minutes and may not involve the participation of virus particles. Virology 186: 712-724. doi:10.1016/0042-6822(92)90038-Q. PubMed: 1370739. 1370739 10.1016/0042-6822(92)90038-q

[2] Sourisseau M, Sol-Foulon N, Porrot F, Blanchet F, Schwartz O (2007) Inefficient human immunodeficiency virus replication in mobile lymphocytes. J Virol 81: 1000-1012. doi:10.1128/JVI.01629-06. PubMed: 17079292. 17079292 10.1128/JVI.01629-06 PMC1797449

[3] de Witte L, de Vries RD, van der Vlist M, Yüksel S, Litjens M et al. (2008) DC-SIGN and CD150 have distinct roles in transmission of measles virus from dendritic cells to T-lymphocytes. PLOS Pathog 4: e1000049 PubMed: 18421379. 18421379 10.1371/journal.ppat.1000049 PMC2277461

[4] Wu L, KewalRamani VN (2006) Dendritic-cell interactions with HIV: infection and viral dissemination. Nat Rev Immunol 6: 859-868. doi:10.1038/nri1960. PubMed: 17063186. 17063186 10.1038/nri1960 PMC1796806

[5] Greco G, Pal S, Pasqualini R, Schnapp LM (2002) Matrix fibronectin increases HIV stability and infectivity. J Immunol 168: 5722-5729. PubMed: 12023372. 12023372 10.4049/jimmunol.168.11.5722

[6] Cavrois M, Neidleman J, Kreisberg JF, Greene WC (2007) In vitro derived dendritic cells trans-infect CD4 T cells primarily with surface-bound HIV-1 virions. PLOS Pathog 3: e4. doi:10.1371/journal.ppat.0030004. PubMed: 17238285. 17238285 10.1371/journal.ppat.0030004 PMC1779297

[7] Willmon C, Harrington K, Kottke T, Prestwich R, Melcher A et al. (2009) Cell carriers for oncolytic viruses: Fed Ex for cancer therapy. Mol Ther 17: 1667-1676. doi:10.1038/mt.2009.194. PubMed: 19690519. 19690519 10.1038/mt.2009.194 PMC2834999

[8] Murphy SL, Li H, Zhou S, Schlachterman A, High KA (2008) Prolonged Susceptibility to Antibody-mediated Neutralization for Adeno-associated Vectors Targeted to the Liver. Mol Ther 16: 138-145. doi:10.1038/sj.mt.6300334. PubMed: 17955024. 17955024 10.1038/sj.mt.6300334

[9] Haim H, Steiner I, Panet A (2007) Time frames for neutralization during the human immunodeficiency virus type 1 entry phase, as monitored in synchronously infected cell cultures. J Virol 81: 3525-3534. doi:10.1128/JVI.02293-06. PubMed: 17251303. 17251303 10.1128/JVI.02293-06 PMC1866073

[10] Sattentau Q (2008) Avoiding the void: cell-to-cell spread of human viruses. Nat Rev Microbiol 6: 815-826. doi:10.1038/nrmicro1972. PubMed: 18923409. 18923409 10.1038/nrmicro1972

[11] Andreadis S, Lavery T, Davis HE, Le Doux JM, Yarmush ML et al. (2000) Toward a more accurate quantitation of the activity of recombinant retroviruses: alternatives to titer and multiplicity of infection. J Virol 74: 1258-1266. doi:10.1128/JVI.74.3.1258-1266.2000. PubMed: 10627536. 10627536 10.1128/jvi.74.3.1258-1266.2000 PMC111460

[12] O’Doherty U, Swiggard WJ, Malim MH (2000) Human immunodeficiency virus type 1 spinoculation enhances infection through virus binding. J Virol 74: 10074-10080. doi:10.1128/JVI.74.21.10074-10080.2000. PubMed: 11024136. 11024136 10.1128/jvi.74.21.10074-10080.2000 PMC102046

[13] Pais-Correia AM, Sachse M, Guadagnini S, Robbiati V, Lasserre R et al. (2010) Biofilm-like extracellular viral assemblies mediate HTLV-1 cell-to-cell transmission at virological synapses. Nat Med 16: 83-89. doi:10.1038/nm.2065. PubMed: 20023636. 20023636 10.1038/nm.2065

[14] Sherer NM, Jin J, Mothes W (2010) Directional Spread of Surface Associated Retroviruses Regulated by Differential Virus-Cell Interactions. J Virol 84: 3248-3258. 20089647 10.1128/JVI.02155-09 PMC2838107

[15] Sherer NM, Mothes W (2008) Cytonemes and tunneling nanotubules in cell-cell communication and viral pathogenesis. Trends Cell Biol, 18: 414–20. PubMed: 18703335. 18703335 10.1016/j.tcb.2008.07.003 PMC2628975

[16] Dimitrov DS, Willey RL, Sato H, Chang LJ, Blumenthal R et al. (1993) Quantitation of human immunodeficiency virus type 1 infection kinetics. J Virol 67: 2182-2190. PubMed: 8445728. 8445728 10.1128/jvi.67.4.2182-2190.1993 PMC240333

[17] Vassalli JD, Lombardi T, Wohlwend A, Montesano R, Orci L (1986) Direct cell-to-cell transmission of vesicular stomatitis virus. J Cell Sci 85: 125-131. PubMed: 3025232. 3025232 10.1242/jcs.85.1.125

[18] Naldini L, Blömer U, Gage FH, Trono D, Verma IM (1996) Efficient transfer, integration, and sustained long-term expression of the transgene in adult rat brains injected with a lentiviral vector. Proc Natl Acad Sci USA 93: 11382-11388. doi:10.1073/pnas.93.21.11382. PubMed: 8876144. 8876144 10.1073/pnas.93.21.11382 PMC38066

[19] Naldini L, Blömer U, Gallay P, Ory D, Mulligan R et al. (1996) In vivo gene delivery and stable transduction of nondividing cells by a lentiviral vector. Science 272: 263-267. doi:10.1126/science.272.5259.263. PubMed: 8602510. 8602510 10.1126/science.272.5259.263

[20] Mátrai J, Chuah MK, Vandendriessche T (2010) Recent advances in lentiviral vector development and applications. Mol Ther 18: 477-490. doi:10.1038/mt.2009.319. PubMed: 20087315. 20087315 10.1038/mt.2009.319 PMC2839421

[21] Montini E, Cesana D, Schmidt M, Sanvito F, Bartholomae CC et al. (2009) The genotoxic potential of retroviral vectors is strongly modulated by vector design and integration site selection in a mouse model of HSC gene therapy. J Clin Invest 119: 964-975. doi:10.1172/JCI37630. PubMed: 19307726. 19307726 10.1172/JCI37630 PMC2662564

[22] Verhoeyen E, Cosset FL (2009) Engineering the surface glycoproteins of lentiviral vectors for targeted gene transfer. CSHProtoc 2009: pdb.top59 10.1101/pdb.top59 20147256

[23] Pan YW, Scarlett JM, Luoh TT, Kurre P (2007) Prolonged adherence of human immunodeficiency virus-derived vector particles to hematopoietic target cells leads to secondary transduction in vitro and in vivo. J Virol 81: 639-649. doi:10.1128/JVI.01089-06. PubMed: 17035328. 17035328 10.1128/JVI.01089-06 PMC1797443

[24] O’Neill LS, Skinner AM, Woodward JA, Kurre P (2010) Entry kinetics and cell-cell transmission of surface-bound retroviral vector particles. J Gene Med 12: 463-476. doi:10.1002/jgm.1458. PubMed: 20440757. 20440757 10.1002/jgm.1458 PMC2864923

[25] Pizzato M, Blair ED, Fling M, Kopf J, Tomassetti A et al. (2001) Evidence for nonspecific adsorption of targeted retrovirus vector particles to cells. Gene Ther 8: 1088-1096. doi:10.1038/sj.gt.3301494. PubMed: 11526456. 11526456 10.1038/sj.gt.3301494

[26] Sharma S, Miyanohara A, Friedmann T (2000) Separable mechanisms of attachment and cell uptake during retrovirus infection. J Virol 74: 10790-10795. doi:10.1128/JVI.74.22.10790-10795.2000. PubMed: 11044124. 11044124 10.1128/jvi.74.22.10790-10795.2000 PMC110954

[27] Blömer U, Gruh I, Witschel H, Haverich A, Martin U (2005) Shuttle of lentiviral vectors via transplanted cells in vivo. Gene Ther 12: 67-74. doi:10.1038/sj.gt.3302384. PubMed: 15385952. 15385952 10.1038/sj.gt.3302384

[28] Haas DL, Case SS, Crooks GM, Kohn DB (2000) Critical factors influencing stable transduction of human CD34(+) cells with HIV-1-derived lentiviral vectors. Mol Ther 2: 71-80. doi:10.1006/mthe.2000.0094. PubMed: 10899830. 10899830 10.1006/mthe.2000.0094

[29] Galimi F, Noll M, Kanazawa Y, Lax T, Chen C et al. (2002) Gene therapy of Fanconi anemia: preclinical efficacy using lentiviral vectors. Blood 100: 2732-2736. doi:10.1182/blood-2002-04-1245. PubMed: 12351379. 12351379 10.1182/blood-2002-04-1245

[30] Logan AC, Nightingale SJ, Haas DL, Cho GJ, Pepper KA et al. (2004) Factors influencing the titer and infectivity of lentiviral vectors. Hum Gene Ther 15: 976-988. doi:10.1089/hum.2004.15.976. PubMed: 15585113. 15585113 10.1089/hum.2004.15.976

[31] Sastry L, Xu Y, Johnson T, Desai K, Rissing D et al. (2003) Certification assays for HIV-1-based vectors: frequent passage of gag sequences without evidence of replication-competent viruses. Mol Ther 8: 830-839. doi:10.1016/j.ymthe.2003.08.003. PubMed: 14599817. 14599817 10.1016/j.ymthe.2003.08.003

[32] Wiley RD, Gummuluru S (2006) Immature dendritic cell-derived exosomes can mediate HIV-1 trans infection. Proc Natl Acad Sci U S A 103: 738-743. doi:10.1073/pnas.0507995103. PubMed: 16407131. 16407131 10.1073/pnas.0507995103 PMC1334656

[33] Platt EJ, Kozak SL, Durnin JP, Hope TJ, Kabat D (2010) Rapid dissociation of HIV-1 from cultured cells severely limits infectivity assays, causes the inactivation ascribed to entry inhibitors, and masks the inherently high level of infectivity of virions. J Virol 84: 3106-3110. doi:10.1128/JVI.01958-09. PubMed: 20042508. 20042508 10.1128/JVI.01958-09 PMC2826045

[34] Mazurov D, Ilinskaya A, Heidecker G, Filatov A (2012) Role of O-glycosylation and expression of CD43 and CD45 on the surfaces of effector T cells in human T cell leukemia virus type 1 cell-to-cell infection. J Virol 86: 2447-2458. PubMed: 22171268. 22171268 10.1128/JVI.06993-11 PMC3302266

[35] Thoulouze MI, Alcover A (2011) Can viruses form biofilms? Trends Microbiol 19: 257-262. doi:10.1016/j.tim.2011.03.002. PubMed: 21458997. 21458997 10.1016/j.tim.2011.03.002

[36] Le Rouzic E, Benichou S (2005) The Vpr protein from HIV-1: distinct roles along the viral life cycle. Retrovirology 2: 11. doi:10.1186/1742-4690-2-11. PubMed: 15725353. 15725353 10.1186/1742-4690-2-11 PMC554975

[37] Skinner AM, O’Neill SL, Kurre P (2009) Cellular microvesicle pathways can be targeted to transfer genetic information between non-immune cells. PLOS ONE 4: e6219. doi:10.1371/journal.pone.0006219. PubMed: 19593443. 19593443 10.1371/journal.pone.0006219 PMC2704871

[38] McDonald D, Vodicka MA, Lucero G, Svitkina TM, Borisy GG et al. (2002) Visualization of the intracellular behavior of HIV in living cells. J Cell Biol 159: 441-452. doi:10.1083/jcb.200203150. PubMed: 12417576. 12417576 10.1083/jcb.200203150 PMC2173076

[39] Abela IA, Berlinger L, Schanz M, Reynell L, Günthard HF et al. (2012) Cell-cell transmission enables HIV-1 to evade inhibition by potent CD4bs directed antibodies. PLOS Pathog 8: e1002634 PubMed: 22496655. 22496655 10.1371/journal.ppat.1002634 PMC3320602

[40] Beer C, Pedersen L (2007) Matrix fibronectin binds gammaretrovirus and assists in entry: new light on viral infections. J Virol 81: 8247-8257. doi:10.1128/JVI.00312-07. PubMed: 17522212. 17522212 10.1128/JVI.00312-07 PMC1951278

[41] Mothes W, Sherer NM, Jin J, Zhong P (2010) Virus cell-to-cell transmission. J Virol, 84: 8360-8368. PubMed: 20375157. 20375157 10.1128/JVI.00443-10 PMC2918988

[42] Zhong P, Agosto LM, Ilinskaya A, Dorjbal B, Truong R et al. (2013) Cell-to-cell transmission can overcome multiple donor and target cell barriers imposed on cell-free HIV. PLOS ONE 8: e53138. doi:10.1371/journal.pone.0053138. PubMed: 23308151. 23308151 10.1371/journal.pone.0053138 PMC3538641

[43] Monel B, Beaumont E, Vendrame D, Schwartz O, Brand D et al. (2012) HIV cell-to-cell transmission requires the production of infectious virus particles and does not proceed through env-mediated fusion pores. J Virol 86: 3924-3933. doi:10.1128/JVI.06478-11. PubMed: 22258237. 22258237 10.1128/JVI.06478-11 PMC3302491

[44] Jin J, Sherer NM, Heidecker G, Derse D, Mothes W (2009) Assembly of the murine leukemia virus is directed towards sites of cell-cell contact. PLOS Biol 7: e1000163 PubMed: 19636361. 19636361 10.1371/journal.pbio.1000163 PMC2709449

