# Supplementary figures and images for: Cell- Cell Transmission of VSV-G Pseudotyped Lentivector Particles
Source: PLoS One. 2013 Sep 10;8(9):e74925. doi: 10.1371/journal.pone.0074925 (PMC3769293; doi:10.1371/journal.pone.0074925)

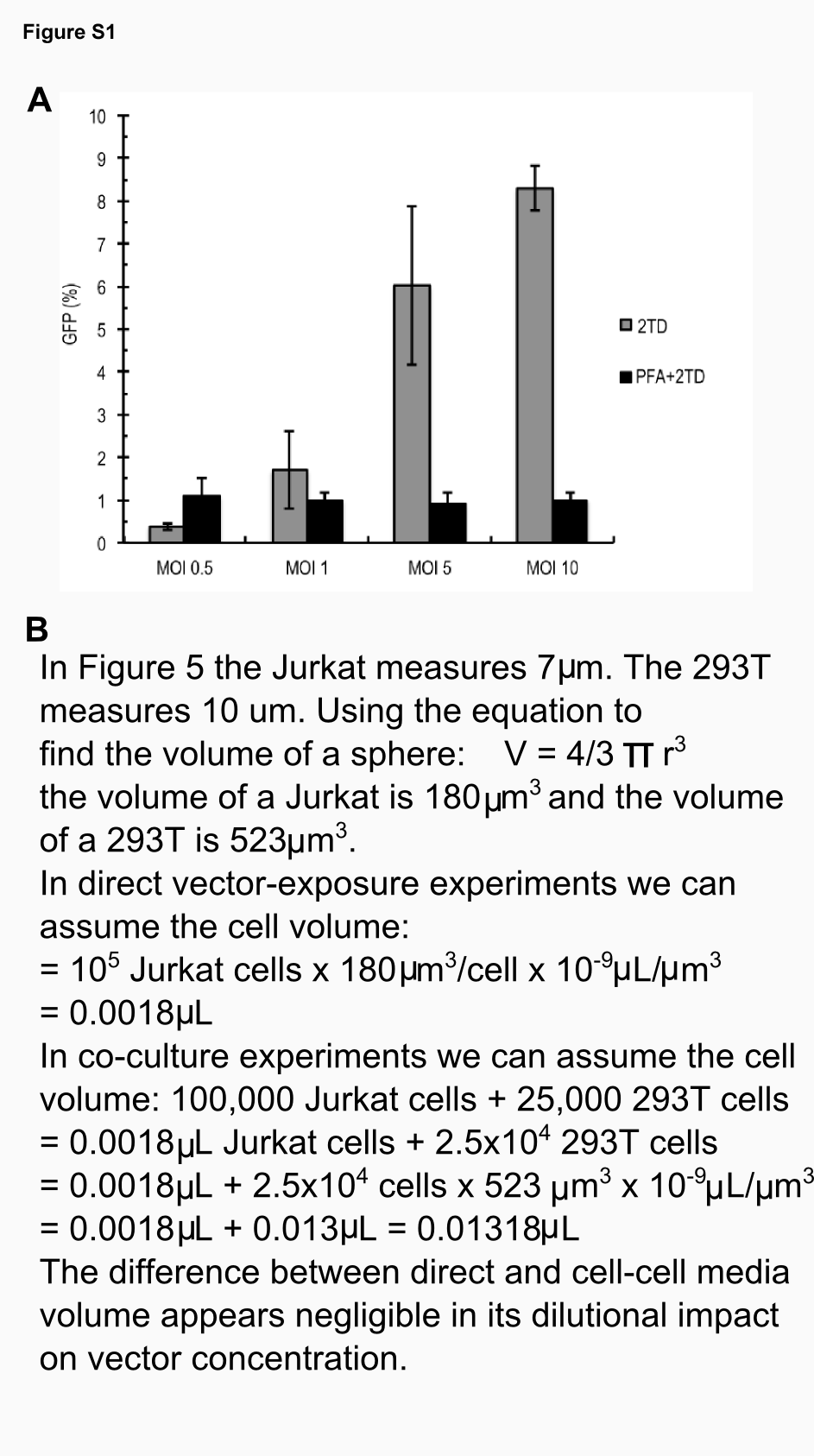

Supplement: Figure S1 — (A) Jurkat cells (1.2x10^6^) were fixed with 4% paraformaldehyde, followed by a 1-hour vector exposure at 4°C in the presence of 8 µg/ml protamine sulfate. Cells were then placed into overnight co-culture with live pre-plated 293T cells (1x10^5^). FACS was performed 48-72 hours later. Secondary transduction was determined in CD45-APC negative GFP+ cells. (B) Mathematical calculations for cell-free and cell-cell culture conditions are shown. (TIF) [file pone.0074925.s001.tif]
